# Response to the Comment on “Effects of Zinc Supplementation on Glycemic Control, Insulin Resistance, Inflammation and Oxidative Stress in Diabetes”

**DOI:** 10.1002/edm2.70376

**Published:** 2026-11-10

**Authors:** Juan Sanchis‐Gimeno, Pablo Nova‐Baeza, Juan Jose Valenzuela‐Fuenzalida, Héctor Gutiérrez‐Espinoza, Alejandro Bruna‐Mejias

**Affiliations:** ^1^ GIAVAL Research Group, Department of Anatomy and Human Embryology, Faculty of Medicine University of Valencia Valencia Spain; ^2^ Departamento de Morfología, Facultad de Medicina Universidad Andrés Bello Santiago Chile; ^3^ Departamento de Ciencias Química y Biológicas, Facultad de Ciencias de la Salud Universidad Bernardo O'Higgins Santiago Chile; ^4^ Faculty of Education Universidad Autónoma de Chile Santiago Chile; ^5^ Universidad Espíritu Santo Samborondón Ecuador

**Keywords:** diabetes mellitus, meta‐analysis as topic, zinc

To the Editor,

We thank Fuentes‐Barría for his comments [1] on our review [2] and welcome the opportunity to clarify its interpretation.

“Glycemic control” identifies an outcome domain assessed, not a claim of improvement. Fasting glucose and glycated haemoglobin were assessed without significant improvements [2]. Naming an assessed outcome in the title should not depend on whether its result is statistically significant.

Section 7 acknowledges these glycemic findings, uncertainty about overall cardiometabolic effects, and the difficulty of isolating zinc‐specific effects. It states that “current evidence remains insufficient to support routine clinical recommendations” [2]. We therefore agree that biochemical changes do not establish patient‐important benefits; this caution is already reflected in the final conclusions.

Sections 2.5 and 5 describe pooled estimates as averages across heterogeneous populations and acknowledge restricted generalizability, co‐interventions, limited study numbers and follow‐up, and incomplete reporting of baseline zinc status [2]. These qualifications do not resolve the limitations, but explain why we share the concern about applying pooled findings to individual patients. Comparisons isolating zinc could provide useful sensitivity analyses; acknowledging co‐interventions does not replace that assessment.

Hu, Zhou and Hattori [3], cited to support excluding multicomponent interventions, address sensitivity to publication bias in meta‐analyses of sparse binary or count data. Their work concerns selective publication, not the influence of co‐interventions. It therefore does not directly support the particular exclusion proposed. This distinction concerns the citation's relevance, not the potential value of the suggested analysis.

We hope these clarifications help distinguish biochemical findings from established clinical benefit.

## Author Contributions

**Juan Sanchis‐Gimeno:** conceptualization, writing – review and editing. **Pablo Nova‐Baeza:** conceptualization. **Juan Jose Valenzuela‐Fuenzalida:** conceptualization, writing – review and editing. **Héctor Gutiérrez‐Espinoza:** writing – review and editing. **Alejandro Bruna‐Mejias:** conceptualization, visualization.

## Funding

The authors have nothing to report.

## Ethics Statement

The authors have nothing to report.

## Consent

The authors have nothing to report.

## Conflicts of Interest

The authors declare no financial conflicts of interest and are coauthors of the review. Group members previously reported eleven letters and commentaries by Héctor Fuentes‐Barría concerning their publications. This history is disclosed for editorial consideration as a potential non‐financial interest; it does not imply improper motives or diminish the scientific points raised.

## Data Availability

No new datasets were generated or analysed for this correspondence.
